# Exploratory Spatial and Temporal Analysis of the Territorial Coexistence of Undernutrition and Excess Weight Among Children Under Five Years of Age in Peru, 2014–2024

**DOI:** 10.3390/ijerph23081033

**Published:** 2026-08-07

**Authors:** Jaime Cesar Prieto-Luna, Luis Alberto Holgado-Apaza, Mixel Luigui Corrido-Ninahuaman, Nestor Antonio Gallegos Ramos, Nelly Jacqueline Ulloa-Gallardo, Dany Dorian Isuiza-Perez, Roxana Madueño-Portilla, Pierre Vidaurre-Rojas, Miguel Angel Valles-Coral

**Affiliations:** 1 Departamento Académico de Ingeniería de Sistemas e Informática, Escuela Profesional de Ingeniería de Sistemas e Informática, Facultad de Ingeniería, Universidad Nacional Amazónica de Madre de Dios, Puerto Maldonado 17001, Peru; mcorridon21-1is@unamad.edu.pe (M.L.C.-N.); nulloa@unamad.edu.pe (N.J.U.-G.); disuiza@unamad.edu.pe (D.D.I.-P.); 2 Amazon Data, Artificial Intelligence and Biodiversitech Research Group, Universidad Nacional Amazónica de Madre de Dios, Puerto Maldonado 17001, Peru; 3 Departamento Académico de Medicina Veterinaria-Zootecnia, Facultad de Ingeniería, Universidad Nacional Amazónica de Madre de Dios, Puerto Maldonado 17001, Peru; rmadueno@unamad.edu.pe; 4 Grupo de Investigación en Inteligencia Artificial, Facultad de Ingeniería de Sistemas e Informática, Universidad Nacional de San Martín, Tarapoto 22201, Peru; pvidaurre@unsm.edu.pe (P.V.-R.); mavalles@unsm.edu.pe (M.A.V.-C.)

**Keywords:** child malnutrition, nutritional surveillance, geospatial analysis, spatial autocorrelation, digital health systems, digital public health

## Abstract

**Highlights:**

**Public health relevance—How does this work relate to a public health issue?**

- Child malnutrition in Peru showed distinct spatial and temporal patterns of undernutrition and excess weight between 2014 and 2024.
- Departmental surveillance records showed geographically differentiated distributions of undernutrition and excess weight at the three cross-sectional time points.

**Public health significance—Why is this work of significance to public health?**

- Global spatial autocorrelation depended on the nutritional indicator and spatial weights matrix, while local LISA classifications were interpreted as unadjusted exploratory findings because multiple comparisons were not formally controlled.
- Median-based descriptive classification identified departments with concurrent high relative undernutrition and excess-weight scores, without establishing temporal expansion or individual-level coexistence.

**Public health implications—What are the key implications or messages for practitioners, policy makers and/or researchers in public health?**

- Spatially explicit surveillance can support the selection of territories for further assessment when coverage, representativeness, spatial scale, and statistical uncertainty are considered.
- Integrating digital nutritional surveillance systems with reproducible geospatial analysis can strengthen evidence-based public health planning and monitoring.

**Abstract:**

The coexistence of undernutrition and excess weight is an important child public health challenge. This study described temporal trends and departmental spatial configurations of wasting, stunting, overweight, and obesity among children under five years of age in Peru during 2014–2024. Analyses used anthropometric assessment records from the Nutritional Status Information System (SIEN), collected in public healthcare facilities and aggregated by department and year. Annual Percentage Change summarized average temporal trends, while observed prevalence maps, Global Moran’s I, Local Indicators of Spatial Association (LISA), and a median-based bivariate classification were applied independently to 2014, 2019, and 2024. Queen contiguity was the primary spatial specification, along with KNN-4 sensitivity analysis. Wasting and obesity increased on average, stunting decreased, and overweight showed no significant long-term trend. Global autocorrelation was most consistent for wasting, whereas findings for excess-weight indicators were sensitive to the weights matrix. LISA identified local spatial classifications at nominal *p* < 0.05; however, these findings were exploratory because no multiple-testing correction was applied. The bivariate classification identified two, four, and three departments with concurrent high relative undernutrition and excess-weight scores in 2014, 2019, and 2024, respectively. These record-based findings show territorial heterogeneity within SIEN but do not represent population prevalence, causal geographic effects, or an integrated spatiotemporal process.

## 1. Introduction

Malnutrition remains one of the leading global public health challenges due to its effects on child survival, growth, and development [1]. This condition encompasses multiple nutritional manifestations, including undernutrition, overweight, and obesity, whose coexistence within the same population is recognized as the double burden of malnutrition [2,3]. In early childhood, undernutrition is primarily expressed as wasting and stunting, whereas excess weight increases the risk of non-communicable diseases from an early age [4,5].

The magnitude of this problem remains substantial. Approximately 45% of deaths among children under five years of age are associated with undernutrition [6,7]. In 2023, an estimated 148 million children were affected by stunting, 45 million by wasting, and more than 37 million by overweight or obesity [8]. The coexistence of nutritional deficits and excesses is particularly evident in Latin America, where progressive reductions in child undernutrition occur alongside sustained increases in overweight and obesity [9,10]. As a result, many countries in the region are experiencing a nutritional transition characterized by heterogeneous patterns of malnutrition and widening territorial health inequalities.

In Peru, chronic child undernutrition has declined over the past decade; however, its distribution remains uneven across territories. The highest prevalences persist in rural, Andean, and Amazonian areas, where limited access to health services, unfavorable socioeconomic conditions, and geographic barriers converge [11]. In contrast, childhood overweight and obesity continue to increase, particularly in urban and coastal areas [12,13]. These differences reflect the coexistence of multiple forms of malnutrition across the national territory and pose important challenges for the planning of targeted nutritional interventions.

The observed disparities are driven by structural determinants that simultaneously influence child nutritional status. Transformations in food systems have increased the availability and consumption of ultra-processed foods, contributing to the rise in excess weight [14]. At the same time, food insecurity, poverty, and socioeconomic inequalities continue to limit access to healthy diets and contribute to the persistence of undernutrition among vulnerable populations [15,16]. These conditions are particularly relevant in territories characterized by marked social and geographic inequalities, where the capacity of health systems to respond is more limited [17].

Spatial characterization of child malnutrition has received increasing attention in Latin America. In Brazil, spatial autocorrelation techniques and geographic analyses have been applied to identify territorial clusters of child undernutrition and excess weight, revealing heterogeneous distributions across regions and population groups [18,19]. Similarly, studies conducted in Colombia, Argentina, and Ecuador have examined the spatial distribution of child malnutrition and its relationship with territorial and socioeconomic determinants using geospatial and ecological approaches [20,21,22,23,24]. However, most available spatial analyses have focused on cross-sectional estimates or short observation periods and have generally examined nutritional indicators separately. Consequently, evidence remains limited on how long-term trends in undernutrition and excess weight correspond to departmental spatial configurations and where both conditions coexist territorially within routine nutritional surveillance data.

The growing availability of digital health surveillance systems has expanded opportunities for population-level analyses of complex public health problems. Digital public health integrates information technologies, data analytics, and computational tools to strengthen epidemiological surveillance and support data-driven decision-making [25,26]. Digital surveillance systems enable the continuous monitoring of health indicators, the identification of emerging territorial patterns, and the planning of interventions across different levels of the health system [27]. In addition, reproducible data science approaches facilitate the integration of large datasets, geospatial analysis, and methodological transparency in large-scale population studies [28,29].

SIEN is a national nutritional surveillance system that compiles anthropometric assessments conducted in Peru’s public healthcare facilities. However, longitudinal SIEN records have rarely been analyzed within a common framework integrating indicator-specific temporal trends, departmental spatial configurations at defined time points, and the territorial coexistence of undernutrition and excess weight. This evidence gap limits understanding of how contrasting nutritional profiles are distributed among departments and how their configurations differ across the surveillance period. Addressing it requires combining record-based temporal summaries with exploratory spatial methods while distinguishing descriptive comparisons from formal spatiotemporal modeling and nationally representative inference.

To address this gap, this study examined temporal trends, spatial configurations, and territorial coexistence of undernutrition and excess weight using SIEN anthropometric assessments of children under five recorded in Peruvian public healthcare facilities from 2014 to 2024. Records were aggregated by department and year, with 25 departmental-level units constituting the spatial scale. APC summarized average temporal trends, whereas choropleth maps, Global Moran’s I, LISA, and median-based bivariate classification characterized distributions, spatial dependence, local associations, and territorial coexistence in 2014, 2019, and 2024. These components were complementary but estimated separately; therefore, comparisons described temporal trends and cross-sectional spatial configurations without modeling spatiotemporal interactions. This framework addresses the stated gap while supporting national nutritional surveillance and informing further population-based and contextual assessment for evidence-based public health planning.

## 2. Materials and Methods

### 2.1. Study Design and Data Source

This ecological study based on routine nutritional surveillance records applied separate but complementary temporal and exploratory spatial analyses using secondary SIEN data. SIEN continuously records anthropometric assessments performed in public healthcare facilities across Peru [30]. Accordingly, the study population comprised healthcare assessments captured by SIEN rather than a probability sample of all Peruvian children.

The temporal component summarized national trends over 2014–2024, whereas spatial analyses described departmental configurations independently for 2014, 2019, and 2024. Cross-sectional comparisons were descriptive and did not estimate temporal spatial dependence, department-specific trajectories, or space–time interaction.

Because SIEN records assessments performed in public healthcare facilities, record-based prevalence may reflect geographic differences in nutritional status, access, attendance, use of private or social-security services, surveillance coverage, and reporting completeness. The findings therefore describe patterns within SIEN and are not nationally representative population estimates.

### 2.2. Study Area and Spatial Unit of Analysis

**Figure 1:**
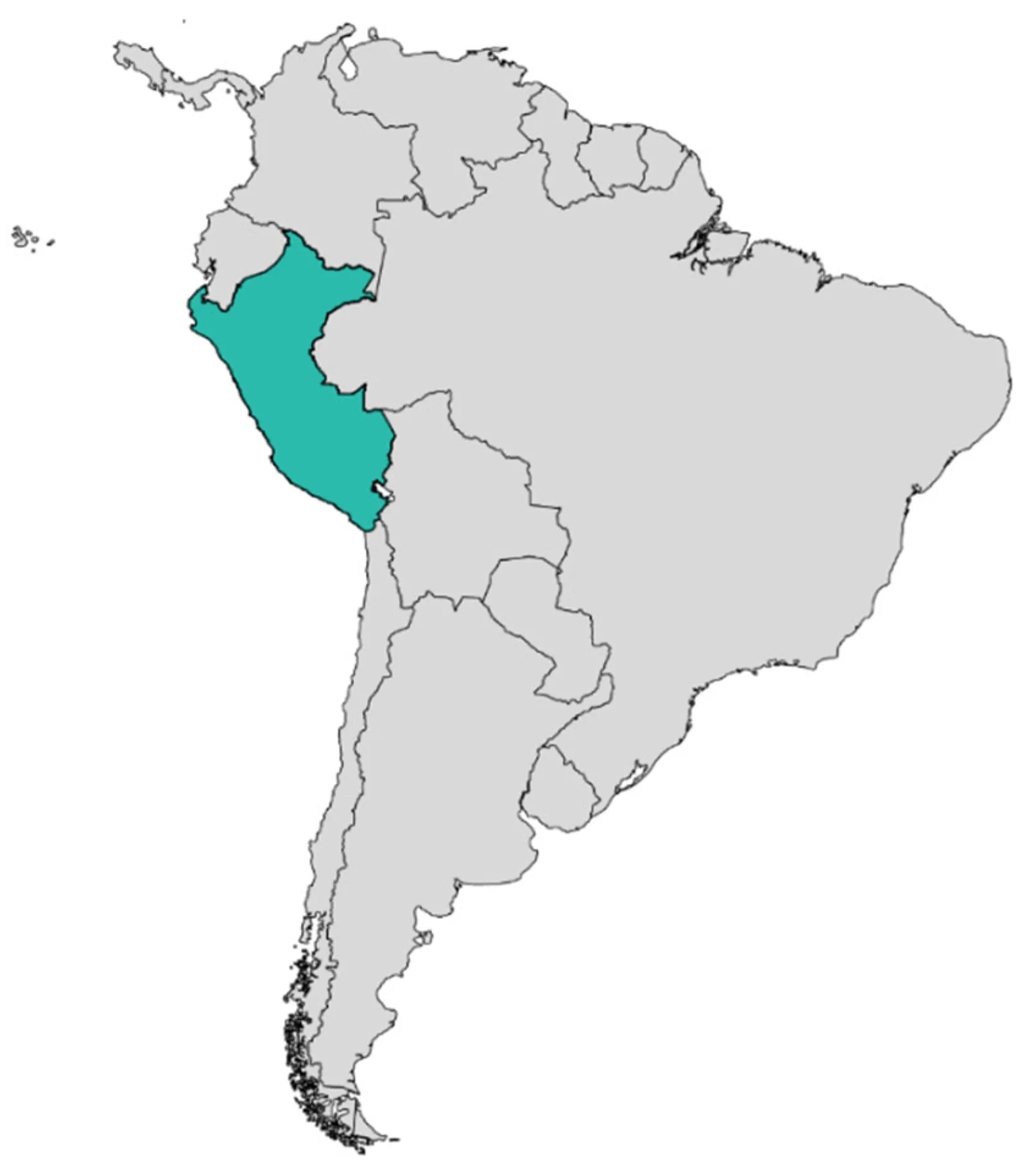
Geographic location of Peru within the South American context. Peru (study area) is highlighted in green.

The study was conducted in Peru, a South American country with a land area of 1,285,216 km^2^, administratively divided into 24 departments and one constitutional province (Figure 1). The country comprises three natural regions (coast, highlands, and Amazon), characterized by marked geographic, demographic, socioeconomic, and healthcare access differences [31].

Spatial analyses used 24 departments and the Constitutional Province of Callao (n = 25). This common scale was selected because the number of districts contributing SIEN records varied across years, preventing a stable district-level panel for 2014–2024. Departmental aggregation improved denominator stability and territorial comparability but reduced the effective spatial sample size, increased sensitivity to the weights matrix, and may conceal within-department heterogeneity. Results cannot be extrapolated to provinces or districts and remain subject to the modifiable areal unit problem.

### 2.3. Study Population and Record Selection

The study population comprised anthropometric assessment of children under five years of age recorded during public healthcare visits between 2014 and 2024. The record-level unit was one assessment, not a verified unique child. A child could contribute multiple records on different dates, including within the same year; these records were retained because no stable identifier was available to construct a unique-child cohort. Exact duplicate rows were removed, but repeated visits were not considered duplicates. Consequently, estimates may disproportionately represent children with more frequent public-service contact.

Records were eligible when they corresponded to children aged 0–59 months and contained valid information on sex, age, weight, height, year of assessment, and department of care. Records were excluded when they were exact duplicates, lacked information required to calculate the nutritional indicators, contained invalid geographic data, or produced anthropometric Z-scores outside the World Health Organization (WHO) plausibility ranges.

**Figure 2:**
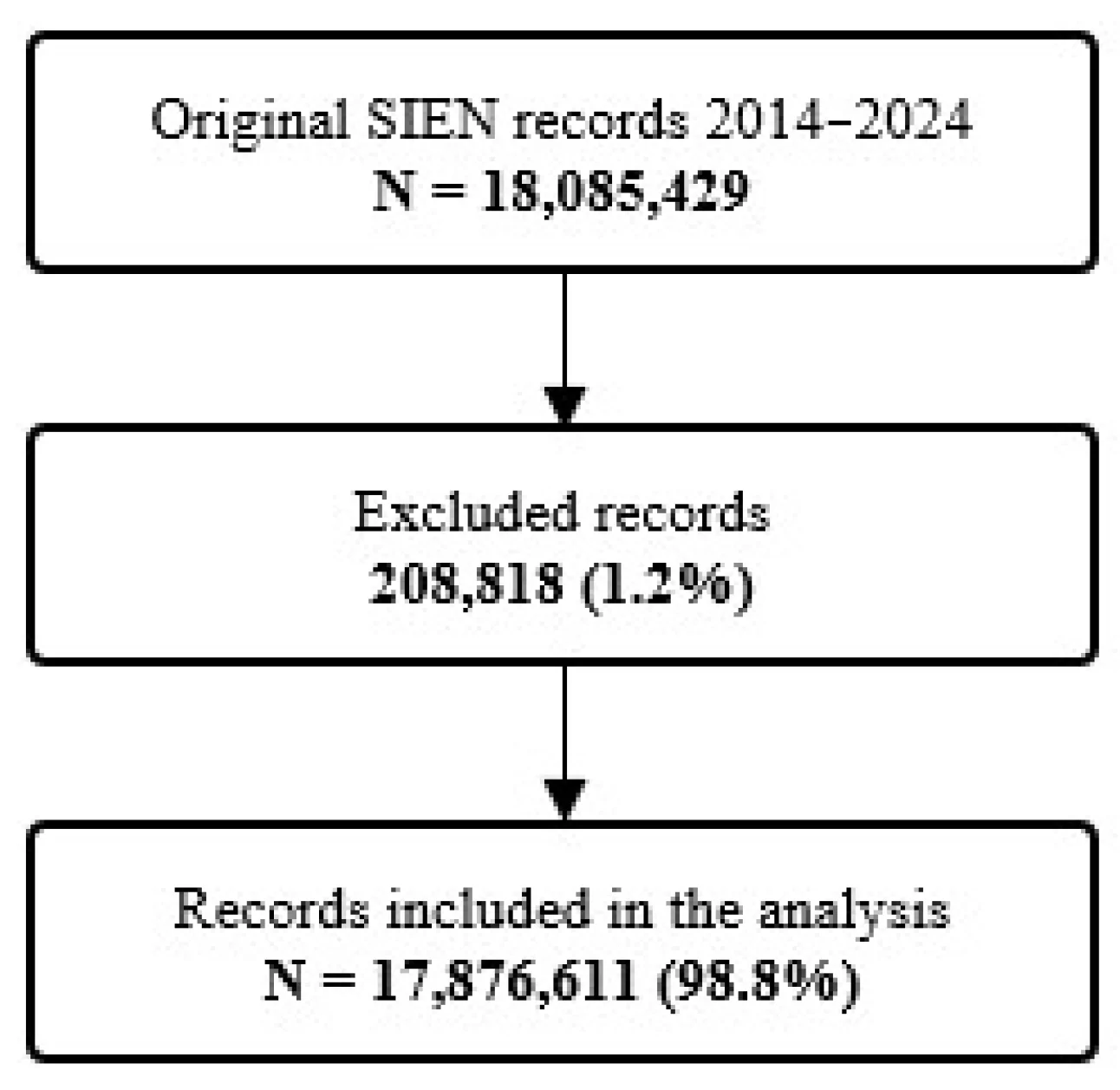
Flow diagram of SIEN anthropometric record selection, Peru, 2014–2024.

The record selection process was documented using a flowchart adapted from the STROBE recommendations (Figure 2). Of the 18,085,429 records retrieved from SIEN, 208,818 records (1.2%) were excluded during data cleaning, resulting in a final analytical dataset of 17,876,611 records.

Valid assessments were aggregated by department of care and year. For each outcome, the numerator was the number meeting the diagnostic definition and the denominator was the number with its relevant valid Z-score: WHZ for wasting, overweight, and obesity, and HAZ for stunting. Department of care may differ from residence; referral and cross-border attendance may therefore introduce spatial misclassification. The resulting prevalences are record-based surveillance measures rather than proportions of unique children.

### 2.4. Nutritional Indicators

The study variables included the prevalences of wasting, stunting, overweight, and obesity, stratified by year, sex, and department.

Nutritional status was assessed using weight-for-height Z-scores (WHZ) and height-for-age Z-scores (HAZ), based on WHO Child Growth Standards and the LMS method [32]. Wasting was defined as WHZ < −2, stunting as HAZ < −2, overweight as +2 < WHZ ≤ +3, and obesity as WHZ > +3, according to WHO Child Growth Standards [33,34]. Overweight and obesity were therefore mutually exclusive.

Given the ecological nature of the study, the territorial coexistence of undernutrition and excess weight was interpreted based on the simultaneous distribution of nutritional deficit indicators (wasting and stunting) and excess weight indicators (overweight and obesity) within the same geographic units. This approach allows the examination of territorial patterns of population-level coexistence without inferring the simultaneous occurrence of both conditions in specific individuals or households [3,5].

### 2.5. Data Processing and Reproducibility

Data processing and analysis were conducted in Python (3.13.2) using a reproducible workflow. Annual datasets corresponding to the 2014–2024 period were integrated into a consolidated and standardized database. Data management and numerical operations used pandas (3.0.3) and NumPy (2.4.6); geospatial processing and spatial statistics used GeoPandas (1.1.3), libpysal (4.14.1), and esda (2.8.2); statistical estimation used statsmodels (0.14.6) and SciPy (1.15.3); and visualizations were generated with Matplotlib (3.10.9) and Seaborn (0.13.2).

Before integration, variable names, data types, character encodings, measurement units, and geographic labels were harmonized across the annual files. Exact duplicates were defined as rows with identical values across all available fields after standardization. Duplicate detection was performed within each annual file and again after consolidation, and only one copy of each exact duplicate was retained. Records corresponding to different assessment dates were not considered duplicates, even when they referred to the same child.

Quality control also identified missing, nonnumeric, or internally inconsistent values for age, sex, weight, height, assessment year, and department. Records with ages outside 0–59 months, nonpositive weight or height values, or geographic information that could not be matched to a valid department were excluded. After these checks, WHZ and HAZ were calculated using the WHO Child Growth Standards and LMS parameters. Values were considered implausible when HAZ was below −6 or above +6, or when WHZ was below −5 or above +5; records outside either range were excluded from the analytical dataset.

The same sequence of validation rules, WHO plausibility thresholds, and exclusion criteria was applied to every annual dataset from 2014 through 2024 through shared processing functions. No year-specific plausibility thresholds were introduced. This standardized procedure ensured that differences among years were not attributable to changes in data-cleaning criteria.

All cleaning, indicator construction, statistical analysis, sensitivity analysis, multiple- testing correction, and figure-generation procedures were implemented through reproducible scripts. The public repository cited in the Data Availability Statement contains the complete executable workflow.

### 2.6. Temporal Trend Analysis

Temporal trends in the prevalence of wasting, stunting, overweight, and obesity were evaluated using the Annual Percentage Change (APC). For each nutritional indicator, annual prevalence estimates were calculated at the national and departmental levels. APC was estimated by fitting a log-linear regression model:

(1) $$log({\hat{p}}_{t})=\alpha +\beta t+\varepsilon_{t}$$

where ${\hat{p}}_{t}$ represents the estimated prevalence in year t, α is the intercept, β is the regression coefficient representing the average annual rate of change, and $\varepsilon_{t}$ is the random error term. APC was calculated as:

(2) $$APC=\left[e^{\beta }-1\right]\times 100$$

Positive APC values indicate increasing prevalence over time, whereas negative values indicate decreasing trends. Statistical significance was established at *p* < 0.05.

APC summarized the average log-linear change over the complete 2014–2024 surveillance period. It was not used to infer constant year-to-year change or identify nonlinear trajectories, pandemic-related discontinuities, or structural breakpoints. Confidence intervals excluding zero were interpreted as evidence of an average increasing or decreasing trend.

### 2.7. Exploratory Spatial Analysis

Spatial analyses used official departmental boundaries and were conducted independently for 2014, 2019, and 2024. Observed record-based prevalences were displayed in choropleth maps. For each indicator-year, case counts, valid denominators, 95% Wilson confidence intervals, and global empirical Bayes (EBS) estimates were retained. EBS reduced instability by shrinking observed rates toward the overall mean according to denominator size; these estimates were not derived from a spatial or spatiotemporal Bayesian model.

First-order row-standardized Queen contiguity was prespecified as the primary spatial weights matrix because it represents direct administrative adjacency. Robustness was assessed using a symmetric row-standardized four-nearest-neighbor matrix derived from departmental representative points in a projected coordinate system [35].

Global spatial autocorrelation was evaluated using global Moran’s I:

(3) $$I=\frac{n}{S_{0}}\frac{\sum_{i=1}^{n}\sum_{j=1}^{n}w_{ij}\left(y_{i}-\bar{y}\right)\left(y_{j}-\bar{y}\right)}{\sum_{i=1}^{n}{\left(y_{i}-\bar{y}\right)}^{2}}$$

where n is the number of departments, $y_{i}$ and $y_{j}$ are the EBS prevalence estimates for departments i and j, $\bar{y}$ is their mean, $w_{ij}$ is the spatial weight between the two departments, and $S_{0}=\sum_{i}\sum_{j}w_{ij}$. Positive values indicate spatial proximity between departments with similar prevalence levels, negative values indicate dissimilarity among neighboring departments, and values near zero indicate limited evidence of global spatial autocorrelation.

Local spatial associations were examined using Local Indicators of Spatial Association:

(4) $$I_{i}=\frac{y_{i}-\bar{y}}{m_{2}}\sum_{j=1}^{n}w_{ij}\left(y_{j}-\bar{y}\right)$$

where

(5) $$m_{2}=\frac{1}{n}\sum_{i=1}^{n}{\left(y_{i}-\bar{y}\right)}^{2}$$

High–High and Low–Low categories represented local associations among similarly high or low EBS prevalence estimates, whereas High–Low and Low–High categories represented local spatial outliers [36]. Statistical significance for Global Moran’s I and LISA was evaluated using 9999 random permutations. Global Moran’s I was estimated under both spatial weights matrices, whereas LISA classifications were based on the primary Queen specification. Sensitivity was assessed by comparing the direction, magnitude, and significance of global estimates and the agreement of local classifications between matrices.

For LISA, local classifications were evaluated using nominal *p* < 0.05 within each indicator-year analysis. Because 25 departmental tests were conducted for every indicator-year combination and no multiple-testing correction was applied, the resulting classifications were treated as exploratory and hypothesis-generating rather than confirmatory evidence of local spatial association.

The spatial procedures were exploratory and did not estimate covariate-adjusted relative risks, department-specific random effects, causal geographic effects, temporal dependence, or area–time interaction. Consequently, differences across years were interpreted descriptively rather than as statistically tested persistence, emergence, disappearance, or expansion.

## 3. Results

### 3.1. Temporal Trends in Nutritional Indicators

The analytical dataset comprised 17,876,611 valid anthropometric records from children under five years of age who attended public health facilities in Peru between 2014 and 2024.

Wasting prevalence increased overall from 1.42% in 2014 to 1.62% in 2024, corresponding to an APC of +2.76% (95% CI: 0.6–4.9). Although prevalence remained higher among boys, the average increase was greater among girls (APC: +3.07%; 95% CI: 0.7–5.5) than boys (APC: +2.53%; 95% CI: 0.5–4.5). Total prevalence declined in 2016, increased progressively from 2018, reached its highest value in 2023 (1.75%), and decreased to 1.62% in 2024 (Table 1).

Stunting prevalence declined from 19.11% in 2014 to 16.02% in 2024, corresponding to a statistically significant average annual decrease (APC: −2.12%; 95% CI: −2.8 to −1.4). Prevalence remained consistently higher among boys, who showed a slightly greater decline (APC: −2.25%; 95% CI: −2.9 to −1.6) than girls (APC: −1.95%; 95% CI: −2.8 to −1.1). Total prevalence decreased steadily until reaching its lowest value in 2021 (15.05%), followed by a moderate increase between 2022 and 2024. Nevertheless, the 2024 prevalence remained 3.09 percentage points below the 2014 level (Table 2).

Overweight prevalence fluctuated without a statistically significant long-term trend. Although prevalence was lower in 2024 (6.31%) than in 2014 (6.45%), the estimated APC was +0.06% (95% CI: −1.0 to 1.2). Prevalence remained consistently higher among boys, while sex-specific trends were also nonsignificant for boys (APC: +0.05%; 95% CI: −1.0 to 1.1) and girls (APC: +0.07%; 95% CI: −1.1 to 1.3). Total prevalence increased from 6.00% in 2015 to 6.81% in 2021, declined to 5.74% in 2023, and rose to 6.31% in 2024 (Table 3).

Obesity prevalence increased from 1.57% in 2014 to 1.89% in 2024, corresponding to a statistically significant APC of +3.42% (95% CI: 0.8–6.1). Prevalence remained consistently higher among boys, although the average increase was slightly greater among girls (APC: +3.53%; 95% CI: 0.7–6.4) than boys (APC: +3.35%; 95% CI: 0.9–5.9). Total prevalence rose markedly in 2020 and reached its highest value in 2021 (2.21%). It subsequently declined to 1.70% in 2023 before increasing to 1.89% in 2024, remaining below the 2021 peak (Table 4).

### 3.2. Spatial Distribution of Nutritional Indicators

**Figure 3:**
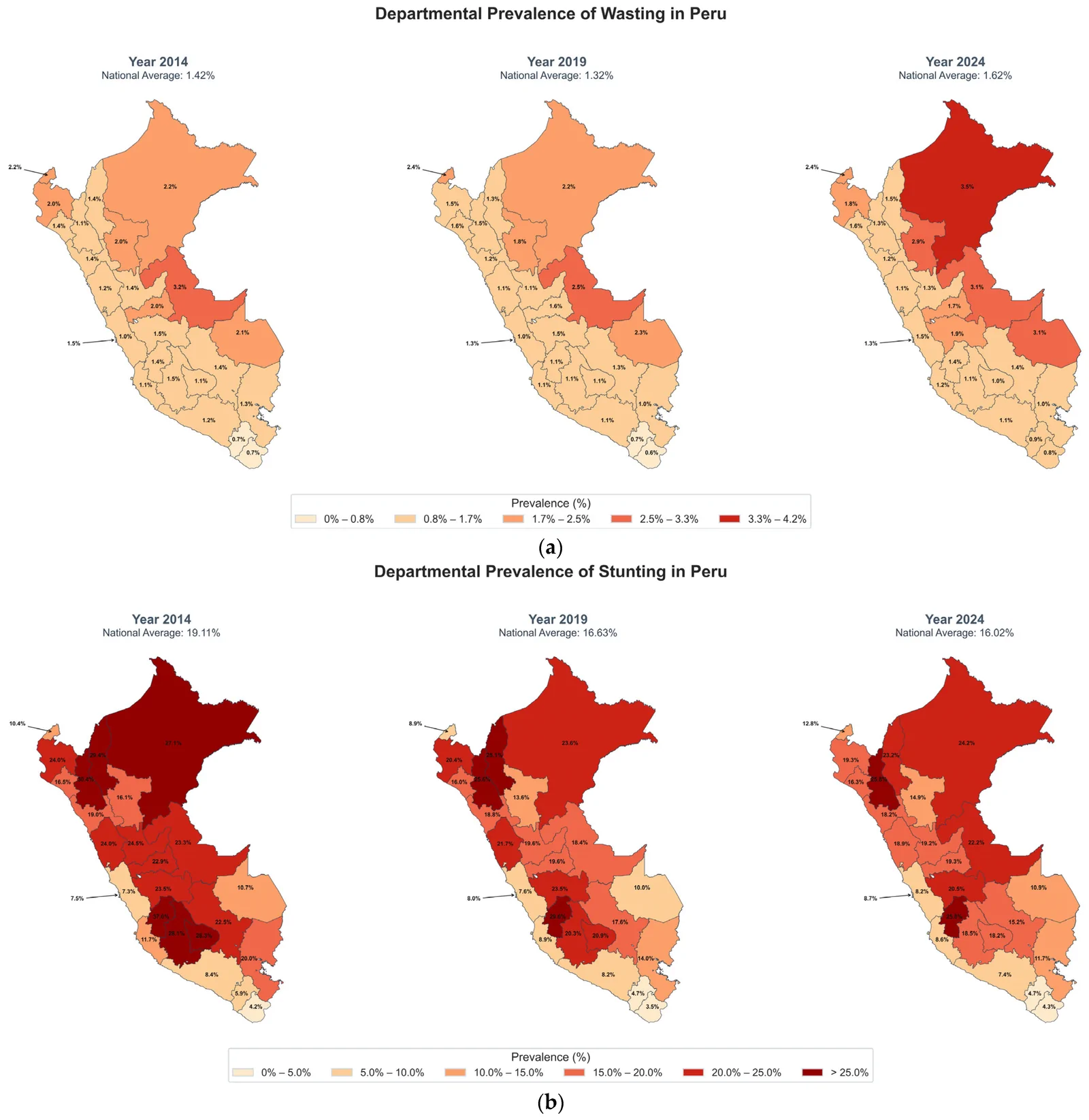
Observed departmental prevalence of (**a**) wasting and (**b**) stunting among children under five years of age in Peru, 2014, 2019, and 2024.

**Figure 4:**
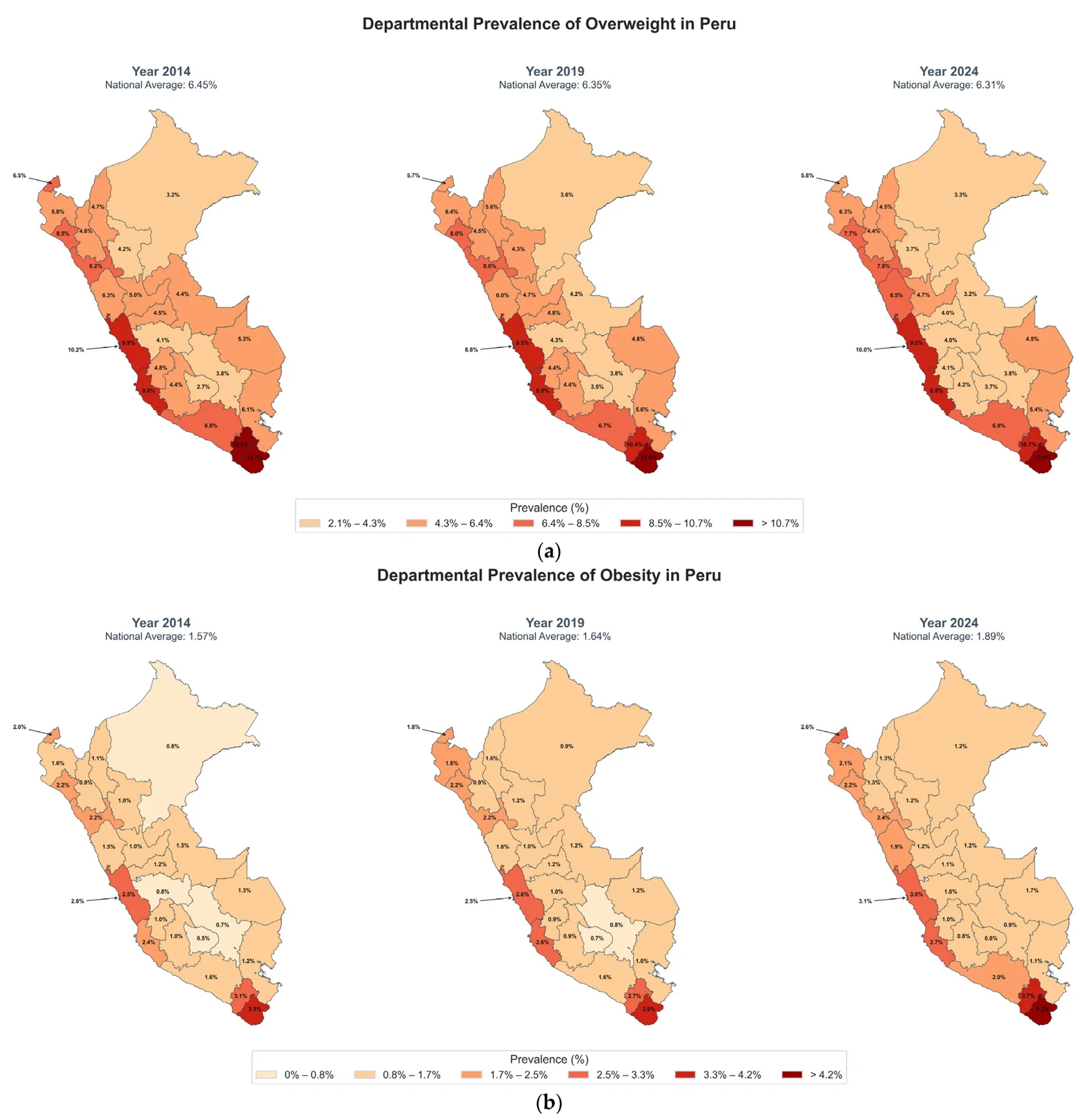
Observed departmental prevalence of (**a**) overweight and (**b**) obesity among children under five years of age in Peru, 2014, 2019, and 2024.

The spatial distribution of malnutrition indicators revealed marked territorial heterogeneity throughout the study period, with distinct patterns observed between undernutrition and excess weight indicators (Figure 3 and Figure 4).

Wasting showed a recurrent concentration of higher observed prevalence in Amazonian departments (Figure 3a). In 2014, the highest values were recorded in Ucayali (3.25%), Loreto (2.23%), Tumbes (2.20%), and Madre de Dios (2.14%), compared with the national prevalence of 1.42%. In 2019, Ucayali (2.55%), Tumbes (2.36%), Madre de Dios (2.32%), and Loreto (2.18%) remained above the national value of 1.32%. By 2024, the highest prevalences occurred in Loreto (3.53%), Ucayali (3.14%), Madre de Dios (3.07%), San Martín (2.87%), and Tumbes (2.40%), while the national prevalence was 1.62%. Conversely, Tacna and Moquegua consistently recorded the lowest values at all three time points.

Stunting showed marked territorial heterogeneity, with the highest observed prevalences concentrated in Andean and Amazonian departments (Figure 3b). In 2014, Huancavelica (37.03%), Cajamarca (30.43%), Amazonas (29.37%), Apurímac (28.26%), Ayacucho (28.11%), and Loreto (27.15%) exceeded the national prevalence of 19.11%. In 2019, the highest values remained in Huancavelica (29.59%), Cajamarca (25.57%), Amazonas (25.08%), and Loreto (23.56%), compared with 16.63% nationally. By 2024, Cajamarca (25.81%), Huancavelica (25.77%), Loreto (24.21%), Amazonas (23.20%), and Ucayali (22.20%) recorded the highest prevalences, while the national value was 16.02%. Conversely, Tacna and Moquegua consistently showed the lowest values, followed by other southern coastal departments and Lima.

Excess-weight indicators showed a spatial distribution distinct from that of undernutrition (Figure 4). Overweight prevalence was consistently higher in coastal departments at each time point (Figure 4a). In 2014, the highest values occurred in Tacna (13.70%), Moquegua (10.89%), Callao (10.23%), and Lima (9.88%), compared with the national prevalence of 6.45%. In 2019, Tacna (12.60%), Moquegua (10.42%), Lima (9.48%), and Ica (9.04%) remained above the national value of 6.35%. By 2024, the highest prevalences were recorded in Tacna (12.95%), Moquegua (10.68%), Callao (10.02%), Lima (9.53%), and Ica (8.82%), while the national prevalence was 6.31%. Conversely, Loreto, Ucayali, Apurímac, Cusco, and San Martín showed comparatively lower values.

Obesity showed a spatial distribution similar to that of overweight but with lower prevalence values (Figure 4b). In 2014, the highest prevalences occurred in Tacna (3.52%), Moquegua (3.11%), Callao (2.75%), Lima (2.55%), and Ica (2.42%), compared with the national value of 1.57%. In 2019, Tacna (3.87%), Moquegua (2.68%), Lima (2.59%), Ica (2.58%), and Callao (2.47%) remained above the national prevalence of 1.64%. By 2024, the highest values were recorded in Tacna (4.29%), Moquegua (3.67%), Callao (3.12%), Lima (3.00%), and Ica (2.73%), while the national prevalence was 1.89%. Conversely, Apurímac, Ayacucho, Cusco, Loreto, and Ucayali showed comparatively lower prevalences in all three cross-sectional assessments.

The maps showed record-based prevalence at three cross-sectional time points. Higher wasting values occurred mainly in Amazonian departments, higher stunting values in Andean and Amazonian departments, and higher overweight and obesity values in coastal departments. These patterns describe geographic contrasts among SIEN assessments; they neither establish population-representative risk nor demonstrate spatial persistence.

### 3.3. Global Spatial Autocorrelation of Nutritional Indicators

Global Moran’s I varied by indicator and spatial weights specification (Table 5). Queen contiguity was the primary analysis, and KNN-4 was used to assess sensitivity. Positive values indicate geographic proximity of similar EBS prevalences, but statistical evidence and magnitude depended on the assumed neighborhood relation.

Under Queen contiguity, wasting showed positive global autocorrelation in 2014 (I = 0.374; *p* = 0.002), 2019 (I = 0.371; *p* = 0.003), and 2024 (I = 0.353; *p* = 0.007). It remained significant under KNN-4 in 2014 (I = 0.218; *p* = 0.020), 2019 (I = 0.283; *p* = 0.010), and 2024 (I = 0.192; *p* = 0.040), providing the most consistent evidence of global spatial structure.

For stunting, Queen estimates were nonsignificant in 2014 (I = 0.174; *p* = 0.065) but significant in 2019 (I = 0.266; *p* = 0.017) and 2024 (I = 0.406; *p* = 0.002). Under KNN-4, the corresponding estimates were nonsignificant in 2014 (I = 0.002; *p* = 0.330) and 2019 (I = 0.086; *p* = 0.140), and significant only in 2024 (I = 0.221; *p* = 0.030).

For overweight, Queen estimates were significant in 2014 (I = 0.380; *p* = 0.002), 2019 (I = 0.338; *p* = 0.006), and 2024 (I = 0.391; *p* = 0.003). KNN-4 estimates were substantially lower and nonsignificant in 2014 (I = 0.095; *p* = 0.120), 2019 (I = 0.085; *p* = 0.130), and 2024 (I = 0.107; *p* = 0.110), indicating that the inference was not robust to the neighborhood definition.

For obesity, Queen estimates were significant in 2014 (I = 0.316; *p* = 0.009), 2019 (I = 0.223; *p* = 0.031), and 2024 (I = 0.304; *p* = 0.009). KNN-4 estimates were nonsignificant in 2014 (I = 0.046; *p* = 0.220), 2019 (I = 0.012; *p* = 0.290), and 2024 (I = 0.042; *p* = 0.210). Thus, global obesity clustering was also specification-dependent.

Overall, global spatial evidence was robust for wasting and for stunting in 2024, whereas conclusions for overweight and obesity depended on the weights matrix. The sensitivity analysis therefore limits any general claim of stable global clustering. Global Moran’s I does not identify the departments contributing to the observed spatial pattern; therefore, unadjusted local LISA classifications were subsequently examined as exploratory results.

### 3.4. Local Spatial Patterns

**Figure 5:**
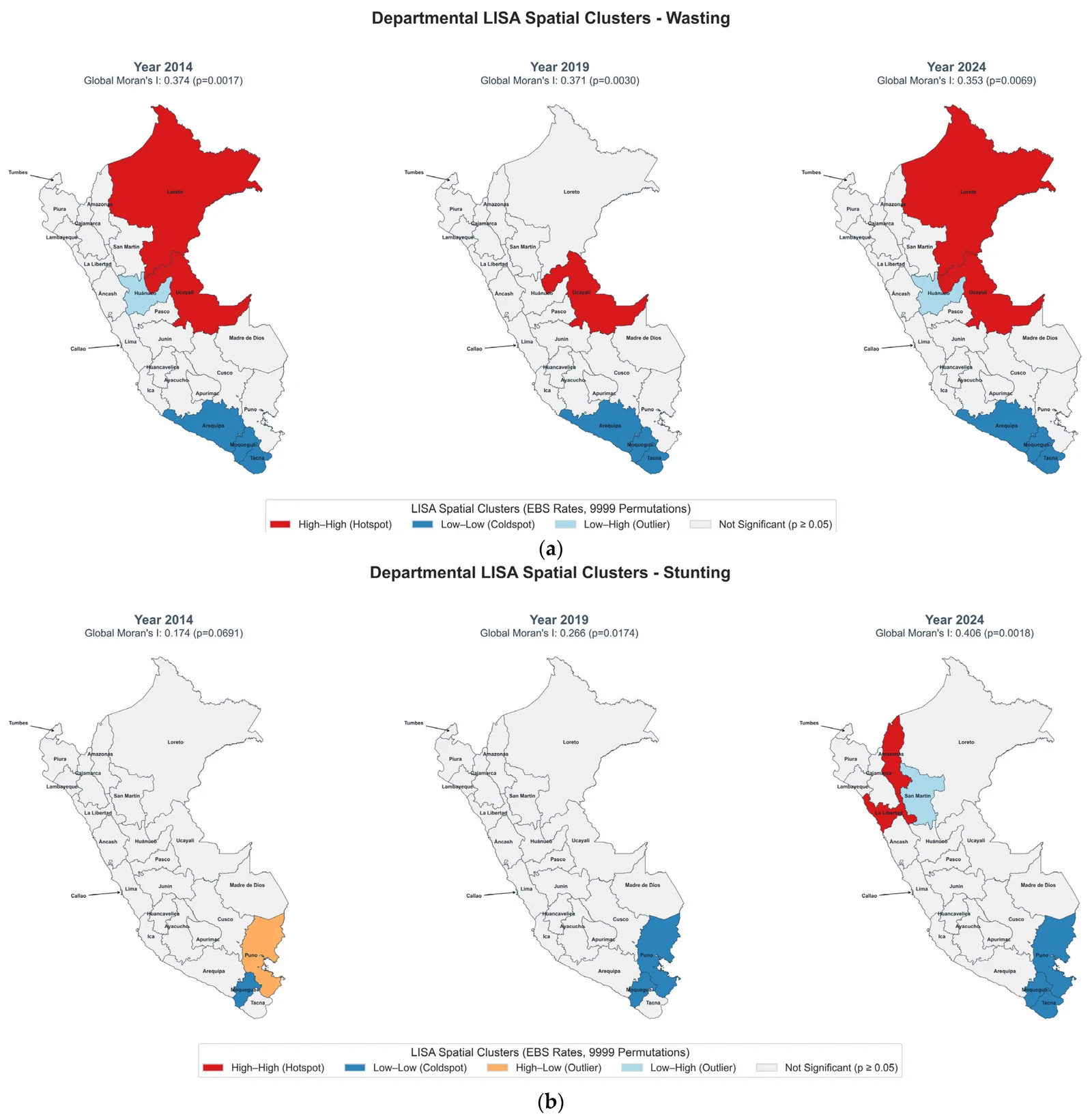
Unadjusted LISA classifications based on EBS prevalence and Queen contiguity for (**a**) wasting and (**b**) stunting among children under five years of age, Peru, 2014, 2019, and 2024. Colored departments satisfy nominal *p* < 0.05. No multiple-testing correction was applied; therefore, the classifications are exploratory.

**Figure 6:**
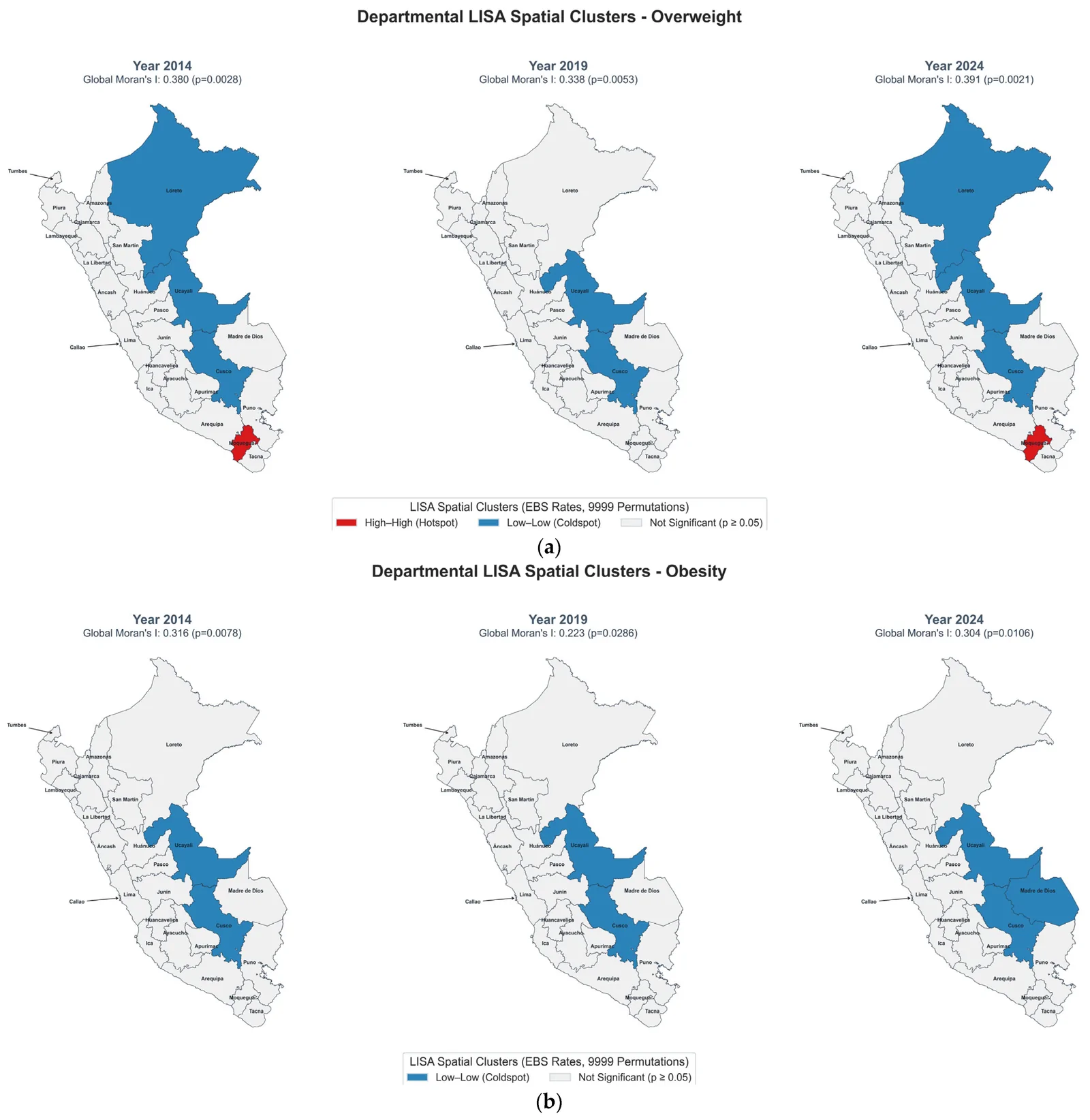
Unadjusted LISA classifications based on EBS prevalence and Queen contiguity for (**a**) overweight and (**b**) obesity among children under five years of age, Peru, 2014, 2019, and 2024. Colored departments satisfy nominal *p* < 0.05. No multiple-testing correction was applied; therefore, the classifications are exploratory.

LISA analyses under Queen contiguity produced local classifications at nominal *p* < 0.05 (Figure 5 and Figure 6). Because 25 departmental tests were performed for each indicator-year and no multiple-testing correction was applied, these classifications were interpreted exclusively as exploratory, hypothesis-generating spatial patterns.

Within this exploratory framework, the LISA results for wasting at nominal *p* < 0.05 (Figure 5a) showed High–High classifications for Ucayali and Loreto in 2014, Ucayali in 2019, and Loreto and Ucayali in 2024. Arequipa, Moquegua, and Tacna showed Low–Low classifications, whereas Huánuco exhibited a Low–High outlier in 2014 and 2024. These unadjusted classifications should be interpreted as exploratory local patterns because multiple comparisons were not controlled.

Regarding stunting, the LISA results at nominal *p* < 0.05 (Figure 5b) showed a High–Low outlier in Puno and a Low–Low classification in Moquegua in 2014; Low–Low classifications in Puno and Moquegua in 2019; and High–High classifications in Amazonas and La Libertad, Low–Low classifications in Puno, Moquegua, and Tacna, and a Low–High outlier in San Martín in 2024. These results represent unadjusted local associations and do not constitute multiplicity-controlled evidence.

Turning to excess-weight indicators, the LISA results for overweight at nominal *p* < 0.05 (Figure 6a) showed a High–High classification in Moquegua in 2014 and 2024 and Low–Low classifications mainly in Ucayali, Cusco, and Loreto. Tacna had the highest observed prevalence but did not satisfy the High–High criterion, illustrating that LISA classifications depend on both departmental and neighboring values. All classifications remain exploratory because no multiple-testing adjustment was performed.

Finally, the LISA results for obesity at nominal *p* < 0.05 (Figure 6b) showed Low–Low classifications for Ucayali and Cusco in 2014 and 2019 and for Madre de Dios, Ucayali, and Cusco in 2024. No High–High classification was detected. These unadjusted findings should be regarded as exploratory because the family-wise set of local tests was not corrected for multiple comparisons.

Overall, the unadjusted maps identified several local configurations at nominal *p* < 0.05. Because multiple comparisons were not controlled, some classifications may represent false-positive findings; therefore, the maps should not be interpreted as confirmed hotspots, coldspots, temporal persistence, emergence, or disappearance.

### 3.5. Territorial Coexistence of Undernutrition and Excess Weight

**Figure 7:**
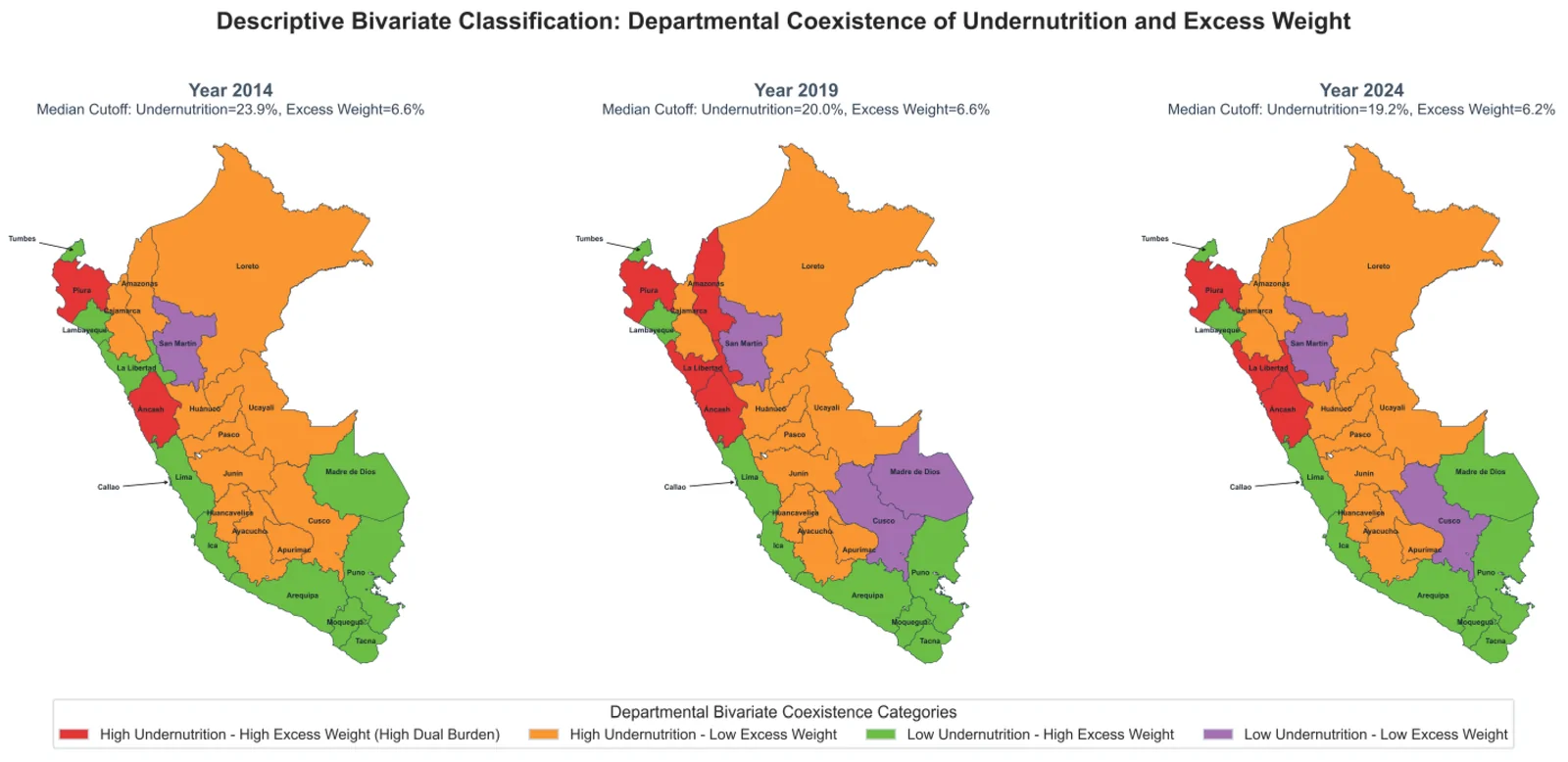
Median-based descriptive classification of departmental undernutrition and excess-weight composite scores among children under five years of age, Peru, 2014, 2019, and 2024.

The median-based descriptive classification compared departmental undernutrition and excess-weight composite scores in 2014, 2019, and 2024 (Figure 7). High and Low categories were defined relative to annual departmental medians rather than clinical thresholds or bivariate LISA significance. Comparisons therefore describe relative cross-sectional configurations and do not test temporal transitions.

In 2014, the median cutoffs for undernutrition and excess weight were 23.91% and 6.62%, respectively. Piura and Ancash were classified as High Undernutrition–High Excess Weight (HH). The High Undernutrition–Low Excess Weight (HL) category included Huancavelica, Cajamarca, Amazonas, Ayacucho, Apurímac, Loreto, Ucayali, Huánuco, Junín, Pasco, and Cusco. Conversely, the Low Undernutrition–High Excess Weight (LH) category comprised most coastal departments, together with Puno and Madre de Dios. San Martín was the only department classified as Low Undernutrition–Low Excess Weight (LL).

In 2019, the median undernutrition cutoff decreased to 20.01%, while the excess-weight cutoff remained at 6.62%. Amazonas, Ancash, Piura, and La Libertad were classified as HH. Nine primarily Andean and Amazonian departments formed the HL category, including Huancavelica, Cajamarca, Loreto, Junín, Apurímac, Ayacucho, Pasco, Ucayali, and Huánuco. The LH category included Lambayeque, Puno, Tumbes, Ica, Arequipa, Callao, Lima, Moquegua, and Tacna. Meanwhile, Cusco, San Martín, and Madre de Dios were classified as LL.

In 2024, the median cutoffs were 19.22% for undernutrition and 6.24% for excess weight. Piura, Ancash, and La Libertad were classified as HH. The HL category comprised ten departments, mainly in the Andean and Amazonian regions, including Loreto, Cajamarca, Huancavelica, Ucayali, Amazonas, Junín, Pasco, Huánuco, Ayacucho, and Apurímac. Ten departments were classified as LH, predominantly along the coast, although this category also included Puno and Madre de Dios. San Martín and Cusco formed the LL category.

The number of High Undernutrition–High Excess Weight departments was two in 2014, four in 2019, and three in 2024. Piura and Ancash were classified in this category at all three time points, La Libertad in 2019 and 2024, and Amazonas only in 2019. Because year-specific median cutoffs were used, these differences indicate changes in relative departmental position and not absolute or statistically tested expansion of the double burden.

## 4. Discussion

This study combined record-based temporal analysis with exploratory departmental spatial analysis of SIEN assessments collected during 2014–2024. Wasting and obesity increased on average, stunting declined, and overweight showed no significant long-term trend. Spatial results varied by indicator and neighborhood definition: wasting showed the most consistent global autocorrelation, whereas evidence for excess-weight indicators was not robust to KNN-4. The median-based classification identified geographically differentiated coexistence profiles but did not show a monotonic expansion of the High–High category.

APC estimates represent average log-linear trajectories over the eleven-year period. They should not be interpreted as constant annual change because the series included fluctuations and the COVID-19 period, during which healthcare utilization, service access, and reporting may have changed. The decline in stunting alongside increases in wasting and obesity is consistent with a heterogeneous nutritional transition, although the surveillance design does not establish population-level trends.

Global and local spatial findings require cautious interpretation. Queen-based Moran estimates indicated broader spatial structure than KNN-4 estimates, demonstrating that conclusions depend partly on the assumed neighborhood relation. In addition, the LISA analyses involved repeated departmental tests without formal multiple-testing correction, which may increase the probability of false-positive local classifications. These findings are therefore best viewed as hypotheses for territorial surveillance rather than adjusted risk areas or evidence of persistent, emerging, or disappearing clusters.

Observed maps showed higher undernutrition values in several Andean and Amazonian departments and higher excess-weight values in several coastal departments. Similar geographic contrasts have been reported in Latin America [19,24,37]. Socioeconomic conditions, healthcare access, food environments, urbanization, and geographic barriers are plausible contextual explanations, but they were not modeled and cannot be inferred from the observed patterns.

The descriptive bivariate classification showed that high relative undernutrition and excess-weight scores could coincide within the same departments. This territorial heterogeneity is consistent with the systematic review and meta-analysis by [38], which documented substantial variation in the prevalence and operational definitions of overlapping forms of malnutrition across Latin America. However, the High–High count changed from two to four and then three, and annual median thresholds also changed. The analysis therefore supports territorial coexistence at selected time points, not progressive expansion, individual double burden, or a formal transition between spatial states.

The principal strengths are the national scope of routine SIEN surveillance, eleven years of records, explicit distinction between assessments and unique children, reproducible processing, and complementary temporal, spatial, sensitivity, and bivariate descriptive analyses. These features support broad territorial monitoring within the surveillance system while preserving the exploratory scope of the design.

SIEN provides continuous routine information that can support territorial monitoring and identify questions requiring further assessment. A comparable application was reported by [39], who used nationwide Brazilian food and nutrition surveillance records to examine temporal trends and social inequalities in child malnutrition, supporting the complementary role of routine surveillance systems alongside population-based evidence. Reproducible geospatial procedures improve transparency; however, surveillance signals should be combined with representative and contextual evidence before intervention or resource-allocation decisions.

Several limitations affect interpretation. SIEN covers public healthcare assessments rather than a representative sample; variation in access, service use, private or social-security care, reporting, and district coverage may influence estimates. Department of care may differ from residence, and repeated visits may overrepresent frequent users. Departmental aggregation yields only 25 spatial units, masks within-department heterogeneity, and is subject to ecological fallacy and the modifiable areal unit problem. Spatial findings were sensitive to the weights matrix, and EBS reduced denominator-related instability without producing adjusted risks or posterior uncertainty. APC did not model nonlinear change, while independent cross-sectional spatial analyses did not estimate temporal dependence or space–time interaction. Future studies should use stable finer-scale coverage where available and hierarchical binomial or beta-binomial spatiotemporal models, including BYM2 spatial effects, flexible temporal terms, territorial covariates, and area–time interactions.

## 5. Conclusions

SIEN assessment records showed divergent average trends and geographically differentiated child malnutrition profiles in Peru during 2014–2024. Wasting and obesity increased, stunting declined, and overweight showed no significant long-term trend. Spatial evidence was strongest and most robust for wasting, while results for other indicators depended more on year and spatial weights. Local LISA classifications were based on unadjusted nominal *p*-values and should therefore be interpreted as exploratory patterns rather than confirmatory evidence of departmental clustering.

Median-based classification identified departments where high relative undernutrition and excess-weight scores coincided, but it did not demonstrate temporal expansion or individual-level double burden. The temporal and spatial analyses were complementary but did not constitute an integrated spatiotemporal model.

These findings can inform surveillance hypotheses and the selection of areas for further assessment. Intervention and resource-allocation decisions should additionally use representative population data, coverage indicators, contextual covariates, and models that quantify spatial and temporal uncertainty.

## Figures and Tables

**Table 1:** Prevalence (%) and Annual Percentage Change (APC) of wasting among anthropometric assessments of children under five years of age by sex, Peru, 2014–2024.

Year	2014	2015	2016	2017	2018	2019	2020	2021	2022	2023	2024	APC (%)	95% CI
Total	1.42	1.48	1.25	1.25	1.26	1.32	1.59	1.61	1.70	1.75	1.62	+2.76	0.6; 4.9
Male	1.59	1.64	1.40	1.39	1.41	1.48	1.76	1.76	1.86	1.91	1.78	+2.53	0.5; 4.5
Female	1.26	1.32	1.09	1.10	1.11	1.16	1.42	1.47	1.54	1.57	1.46	+3.07	0.7; 5.5

**Table 2:** Prevalence (%) and Annual Percentage Change (APC) of stunting among anthropometric assessments of children under five years of age by sex, Peru, 2014–2024.

Year	2014	2015	2016	2017	2018	2019	2020	2021	2022	2023	2024	APC (%)	95% CI
Total	19.11	18.54	17.95	17.46	17.23	16.63	16.56	15.05	15.18	15.82	16.02	−2.12	−2.8; −1.4
Male	20.87	20.23	19.47	19.04	18.89	18.22	17.95	16.49	16.5	17.04	17.13	−2.25	−2.9; −1.6
Female	17.30	16.79	16.36	15.81	15.52	14.97	15.12	13.56	13.83	14.56	14.87	−1.95	−2.8; −1.1

**Table 3:** Prevalence (%) and Annual Percentage Change (APC) of overweight among anthropometric assessments of children under five years of age by sex, Peru, 2014–2024.

Year	2014	2015	2016	2017	2018	2019	2020	2021	2022	2023	2024	APC (%)	95% CI
Total	6.45	6.00	6.03	6.17	6.48	6.35	6.62	6.81	6.30	5.74	6.31	+0.06	−1.0; 1.2
Male	6.89	6.37	6.40	6.56	6.85	6.71	6.97	7.18	6.69	6.14	6.72	+0.05	−1.0; 1.1
Female	6.00	5.62	5.64	5.76	6.10	5.97	6.26	6.43	5.90	5.34	5.89	+0.07	−1.1; 1.3

**Table 4:** Prevalence (%) and Annual Percentage Change (APC) of obesity among anthropometric assessments of children under five years of age by sex, Peru, 2014–2024.

Year	2014	2015	2016	2017	2018	2019	2020	2021	2022	2023	2024	APC (%)	95% CI
Total	1.57	1.39	1.44	1.50	1.67	1.64	2.16	2.21	1.98	1.70	1.89	+3.42	0.8; 6.1
Male	1.77	1.59	1.63	1.70	1.87	1.84	2.38	2.48	2.23	1.93	2.12	+3.35	0.9; 5.9
Female	1.37	1.18	1.23	1.30	1.47	1.42	1.93	1.94	1.72	1.46	1.65	+3.53	0.7; 6.4

**Table 5:** Sensitivity of Global Moran’s I for EBS departmental prevalence under Queen and KNN-4 spatial weights, Peru, 2014, 2019, and 2024.

Indicator	Year	Moran I (Queen)	*p*-Value	Moran I (KNN-4)	*p*-Value	Cluster Concordance (%)
Wasting	2014	0.374	0.002	0.218	0.020	56
	2019	0.371	0.003	0.283	0.010	68
	2024	0.353	0.007	0.192	0.040	56
Stunting	2014	0.174	0.065	0.002	0.330	76
	2019	0.266	0.017	0.086	0.140	68
	2024	0.406	0.002	0.221	0.030	60
Overweight	2014	0.380	0.002	0.095	0.120	56
	2019	0.338	0.006	0.085	0.130	60
	2024	0.391	0.003	0.107	0.110	52
Obesity	2014	0.316	0.009	0.046	0.220	64
	2019	0.223	0.031	0.012	0.290	68
	2024	0.304	0.009	0.042	0.210	64

## Data Availability

The data analyzed in this study are publicly available from the Peruvian Open Data Portal at https://www.datosabiertos.gob.pe/dataset/sien-sistema-de-informaci%C3%B3n-del-estado-nutricional-de-ni%C3%B1os-y-gestantes-per%C3%BA-inscenan (accessed on 20 July 2026). The Python scripts used for data processing, statistical analyses, spatial analyses, and figure generation are openly available at https://github.com/MixelLuiguiCorridoNinahuaman/analysis-spatiotemporal-child-malnutrition-peru-2014-2024 (accessed on 20 July 2026).
